# Mortality Trends in Patients Aged 25 and Older With Comorbid COPD and Diabetes Mellitus in the United States: A CDC WONDER Database Study From 2000 to 2023

**DOI:** 10.1155/carj/3655829

**Published:** 2026-07-27

**Authors:** Muhammad Ahmed, Saba Fatima, Shaheer Bin Shafiq, Khubaib Tariq Mansoor, Fatima Aqsa, Muhammad Abdullah Naveed, Ahila Ali, Harigopal Sandhyavenu, Binish Qureshi, Sivaram Neppala

**Affiliations:** ^1^ Department of Cardiology, Dow Medical College, Dow University of Health Sciences, Karachi, Pakistan, duhs.edu.pk; ^2^ Department of Cardiology, Jinnah Sindh Medical University, Karachi, Pakistan, jsmu.edu.pk; ^3^ Department of Cardiology, UT Health Science Center, San Antonio, Texas, USA, uth.edu

**Keywords:** chronic obstructive pulmonary disease (COPD), diabetes mellitus, gender, mortality, race

## Abstract

**Introduction:**

Diabetes mellitus (DM) and chronic obstructive pulmonary disease (COPD) are among the leading chronic diseases that significantly impact global morbidity and mortality. Growing evidence indicates a bidirectional interplay between metabolic and pulmonary dysfunction. This study aims to identify the demographic and regional trends of DM and COPD‐related mortality in the United States from 2000 to 2023.

**Methodology:**

DM‐ and COPD‐related deaths in the United States from 2000 to 2023 were obtained from the CDC WONDER database using ICD‐10 code E10‐14 for DM and J40‐44 as the underlying cause of death. Results are presented as age‐adjusted mortality rates (AAMR) per 100,000 population, with Joinpoint regression used for trend analysis and to calculate annual percentage change (APC).

**Results:**

A total of 796,757 DM‐ and COPD‐related deaths were recorded. From 2000 to 2008, the AAMR for DM and COPD increased from 11.19 to 14.32, remaining relatively stable until 2018. This was followed by a significant rise to 19.03 in 2021 (APC: 10.57), before declining to 16.28 in 2023 (APC: −9.08). Men demonstrated a higher AAMR (19.28) than women (12.02); however, both experienced decline post‐2021, with rates falling to 20.54 in men (APC: −9.08) and 13.02 in women (APC: −8.33). AAMR increased among all racial groups except Asians and Hispanics. From 2000 to 2021, American Indians had the highest AAMR (30.48). After 2021, AAMR declined across all groups. Rural areas reported higher AAMR (19.02), compared to urban areas (12.85). AAMR in rural areas increased markedly from 2018 to 2020 (13.67). State‐level comparisons revealed substantial variation in AAMR. Oklahoma had the highest rate at 28.79, followed by West Virginia (28.53), Kentucky (26.74), Vermont (23.95), and Mississippi (20.83). In contrast, Hawaii (6.69), Utah (7.33), and Massachusetts (7.99) reported the lowest rates, all below 10. Regionally, the Midwest had the highest average AAMR (16.30).

**Conclusion:**

COPD‐related diabetes mortality rose from 2000 to 2020, highest among men, Non‐Hispanic American Indians, and rural populations, and then declined post‐2021. Findings indicate increased vulnerability during acute events, highlighting the need for targeted interventions.

## 1. Introduction

Diabetes mellitus (DM) and chronic obstructive pulmonary disease (COPD) represent two of the most significant and prevalent chronic diseases. The rise of DM and COPD is a dangerously significant indication to the threat of their impact toward global health. Over 500 million individuals are affected by DM globally, with 37 million cases in the United States, approximately 11% of the population [1, 2]. COPD is defined by long‐term airflow obstruction and chronic respiratory symptoms. A staggering amount of 16 million adults are diagnosed in the United States per annum due to this disease [3]. The economic toll of these diseases is immense: DM costs the U.S. economy $327 billion annually [3], while COPD‐related costs exceed $50 billion [4, 5].

Emerging studies showcase a bidirectional relationship between both the diseases, which raises important public health concerns. Studies indicate that patients with diabetes are at a higher risk for developing COPD; similarly, those suffering from COPD have a higher risk of developing diabetes [6, 7]. The pathogenic processes involved in both conditions show considerable overlap, which includes chronic inflammation, oxidative stress, and endothelial dysfunction, all of which collectively speed up disease progression and worsen the prognosis [8, 9]. Moreover, insulin resistance, sedentary lifestyles, and prolonged use of corticosteroids can further aggravate both metabolic and pulmonary issues [10].

Individuals who suffer from both of these diseases typically face worse health outcomes as compared to those living with just one of these conditions. They have a higher risk of hospitalization, longer stay in hospitals, and elevated mortality rates [11, 12]. Yet, national data on how mortality patterns vary for this comorbid population remain insufficiently studied, especially across diverse demographic and geographic groups.

This study aims to highlight the trends related to DM and COPD in the United States from 1999 to 2023. It will also assess the disparities and variations in these trends by sex, age, race, ethnicity, geographic region, and urbanization level using CDC WONDER database. These insights are essential for guiding targeted public health strategies and improving outcomes for individuals affected by both COPD and DM.

## 2. Methods

### 2.1. Study Design and Cohort

We utilized the CDC WONDER (Centers for Disease Control and Prevention Wide‐Ranging Online Data for Epidemiologic Research) database to obtain death certificates for DM‐ and COPD‐related mortality from 1999 to 2023. We used the International Statistical Classification of Diseases and Related Health Problems‐10th Revision (ICD‐10) codes E10‐14 and J40‐44 to identify deaths due to diabetes and COPD, respectively. The dataset includes cause‐of‐death information from all 50 states and the District of Columbia, focusing on adults aged 25 years and older. The Multiple Cause‐of‐Death Public Use records were used to ensure the inclusion of all cases where DM and COPD were listed as either a direct or underlying cause of death. Since the data were obtained from a publicly accessible database, institutional review board approval was not required. The study adheres to the STROBE (Strengthening the Reporting of Observational Studies in Epidemiology) guidelines for reporting.

### 2.2. Data Extraction

Deaths related to DM and COPD, along with population sizes, were abstracted for the years 2000–2023. We also collected demographic data, including gender and race/ethnicity, as well as geographic information such as state, region, and degree of urbanization (rural vs urban). Race/ethnicity was classified as Non‐Hispanic (NH) White, NH Black or African American, Hispanic or Latino, NH American Indian or Alaskan Native, and NH Asian or Pacific Islander. This classification follows the categories established by the CDC WONDER database and has been used in previous studies [13]. Regions were classified according to U.S. Census Bureau definitions into Northeast, Midwest, South, and West. Urban areas included large central metropolitan, large fringe metropolitan, medium metropolitan, and small metropolitan classes as defined by the CDC WONDER database, while non‐metropolitan areas included micropolitan and noncore regions.

### 2.3. Statistical Analysis

To evaluate nationwide trends in DM‐ and COPD‐related mortality, we calculated the crude and age‐adjusted mortality rate (AAMR) per 100,000 population from 1999 to 2023, stratified by year, gender, race, and urban–rural status with a 95% confidence interval (CI). Additionally, we assessed annual variations in DM‐ and COPD‐related mortality by calculating the annual percentage change (APC) using the Joinpoint Regression Program (Joinpoint V5.0.2, National Cancer Institute), with a 95% CI. This software fits log‐linear regression models to identify significant changes in the AAMR over time. APCs were deemed to be increasing or decreasing if the slope of the mortality trend was significantly different from zero, as determined by two‐tailed *t*‐tests. A *p*‐value of < 0.05 was considered statistically significant.

## 3. Results

**FIGURE 1:**
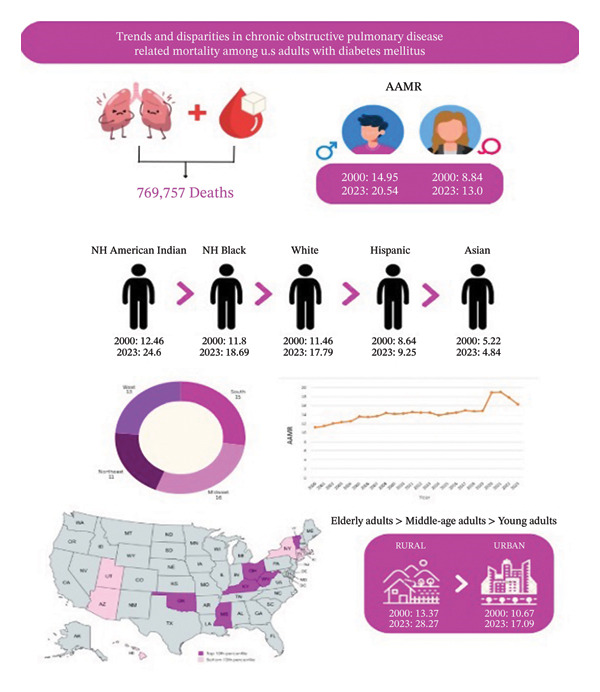
Central illustration depicting trends in demographics and disparities in COPD mortality among adults with diabetes mellitus in the United States from 2000 to 2023. AAMR = age‐adjusted mortality rate; APC = annual percentage change; NH= non‐Hispanic.

Between 1999 and 2023, there were 796,757 recorded deaths in the United States among individuals diagnosed with both COPD and DM (Supporting Table 1). This equates to an AAMR of 14.79 per hundred thousand people. Notably, a vast majority of these deaths 80.78% took place in adults aged 65+. A central illustration summarizing the study’s characteristics and findings is presented in Figure 1.

### 3.1. Annual Trends for COPD With DM‐Related AAMR

A gradual increase in AAMR was seen from 2000 to 2008, rising modestly from 11.19 to 14.32. However, from 2008 to 2018, this AAMR only increased by a mere 0.42, increasing from 14.32 in 2008 to 14.74 in 2018. From 2018 to 2021, the increase in the AAMR was most remarkable increasing from 14.74 in 2018 to 19.03 in 2021 with an APC of 10.57 (95% CI: 7.1, 13.1; *p* < 0.01). From 2021 onward, the AAMR started plummeting, reaching 16.28 in 2023 with an APC of −9.08 (95% CI: −9.0, −12.8; *p* < 0.01) (Supporting Table 3).

### 3.2. COPD With DM‐Related AAMR Stratified by Sex

**FIGURE 2:**
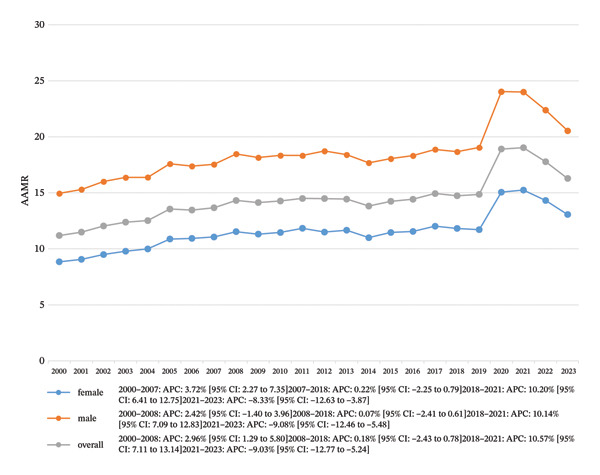
Overall and sex‐stratified COPD‐related age‐adjusted mortality rates per 100,000 adults with diabetes mellitus in the United States from 2000 to 2023. AAMR = age‐adjusted mortality rate; APC = annual percentage change.

When analyzed by sex, men accounted for 54.71% of the total deaths. Throughout the study period, the AAMR among males was consistently higher than among females—19.28 per hundred thousand compared to 12.02 per hundred thousand population. From 2000 to 2008, both men and women saw a slow increase with the AAMR of men increasing from 14.95 in 2000 to 18.47 in 2008 and women increasing from 8.84 in 2000 to 11.53 in 2008. However, from 2008 till 2018, this increase in AAMR became stagnant with males experiencing an increase of 0.20, increasing from 18.47 in 2008 to 18.67 in 2018 with an APC of 0.07 (95% CI: −2.41, 1.40; *p* = 0.97), and females experiencing an AAMR increase of 0.76, increasing from 11.06 in 2007 to 11.82 in 2018 with APC of 0.22 (95% CI: −2.25, 0.79; *p* = 0.72). From 2018 to 2021, there was a remarkable increase in AAMR in both men and women, with men AAMRs experiencing a rise of 5.33 increasing from 18.67 in 2018 to 24.00 in 2021, APC: 10.41(95% CI: 7.1, 12.9; *p* < 0.01) and women AAMRs experienced a rise of 3.42, increasing from 11.82 in 2018 to 15.24 in 2021, APC: 10.20 (95% CI: 6.4, 12.7; *p* < 0.01). From 2021 onward, the AAMR showed a downward trend, with both men and women experiencing significant decreases. For men, the AAMR decreased from 24.00 in 2021 to 20.54 in 2023, and for women, 15.24 in 2021 to 13.06 in 2023, with respective APCs of −9.08 (95% CI: −9.07, −12.45; *p* < 0.01) for men and −8.33 (95% CI: −8.32, −12.63; *p* < 0.01) for women (Supporting Table 2 and Figure 2).

### 3.3. COPD With DM‐Related AAMR Stratified by Age Group

**FIGURE 3:**
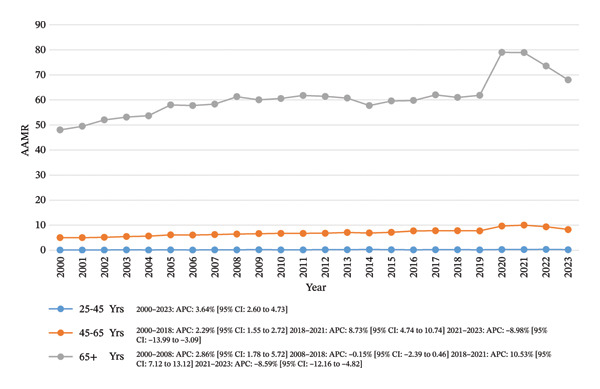
COPD‐related age‐adjusted mortality rates per 100,000 stratified by age groups in adults (≥ 25 years) with diabetes mellitus in the United States, 1999 to 2023. AAMR = age‐adjusted mortality rate; APC = annual percentage change.

Age‐wise, older adults (65+) were disproportionately affected, representing over 80% of deaths. Their AAMR stood at 60.74 per hundred thousand, significantly higher than the 0.24 per hundred thousand seen in adults aged 25–44 and 7.02 in adults aged 45–64. While younger adults aged 25–64 saw only a slight rise in mortality from 2000 to 2023, older adults experienced a steady increase until 2008, followed by a static period till 2018, APC −0.15 (95% CI: −2.39, 0.46; *p* < 0.01). From 2018 to 2021, there was a rapid increase in AAMR from 60.99 in 2018 to 78.92 in 2021 in the older adults aged 65+ with an APC of 10.53 (95% CI: 7.11, 13.12; *p* < 0.01). Though from 2021, the rates plummeted with a AAMR decrease of 10.88, decreasing from 78.92 in 2021 to 68.04 in 2023 with an APC of −8.59 (95% CI: −12.15, −4.81; *p* < 0.01) in 3 years (Supporting Table 8 and Figure 3).

### 3.4. COPD With DM‐Related AAMR Stratified by Race/Ethnicity

**FIGURE 4:**
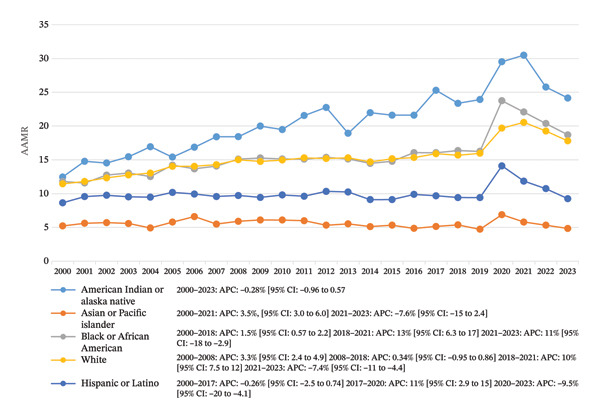
COPD‐related age‐adjusted mortality rates per 100,000 stratified by race in adults with diabetes mellitus in the United States, 2000 to 2023. AAMR = age‐adjusted mortality rate; APC = annual percentage change; NH= non‐Hispanic.

Racial analysis revealed that American Indians or Alaska Natives, Blacks or African Americans, and Whites experienced increasing trends up until 2021, with the American Indian population showing the steepest climb AAMR increasing from 12.46 in 2000 to 30.48 in 2021. In contrast, the Asian and Hispanic population did not experience any significant increases during this period. However, from 2021 onwards, American Indians (30.48–24.15) APC −7.60 (95% CI: −14.88, 2.37; *p* < 0.01), Blacks (22.07–18.69) APC −11.25 (95% CI: −17.76, −2.94; *p* < 0.01), and Whites (20.54–17.79) APC −7.43 (95% CI: −10.70, −4.44; *p* < 0.01) experienced drops in AAMRs (Supporting Table 4 and Figure 4).

### 3.5. COPD With DM‐Related AAMR Stratified by Geographical Regions

**FIGURE 5:**
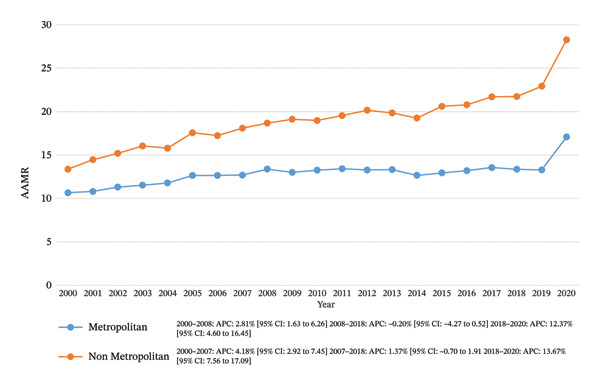
COPD‐related age‐adjusted mortality rates per 100,000 stratified by urbanization in adults (≥ 25 Years) with diabetes mellitus in the United States, 1999 to 2023. AAMR = age‐adjusted mortality rate; APC = annual percentage change.

Looking at geographical distribution based on urbanization, the AAMR of rural region was generally higher than that of urban areas. Rural regions had a higher overall rate (19.02 per hundred thousand) compared to urban ones (12.85 per hundred thousand). However, notable increases were observed in the period from 2018 to 2020 in both urban and rural areas. Urban areas experience an increase of 3.73 in the AAMRs, increasing from 13.36 in 2018 to 17.09 in 2020 with an APC of 12.37 (95% CI: 4.60, 16.45; *p* < 0.01), and rural areas experience a comparatively greater increase with an AAMR increase of 6.53, increasing from 21.74 in 2018 to 28.27 in 2020 with an APC of 13.67 (95% CI: 7.55, 17.09; *p* < 0.01) (Supporting Table 6,8 and Figure 5).

State‐by‐state comparisons showed considerable variation. The State of Oklahoma recorded the highest AAMR at 28.79, followed by West Virginia at 28.53, Kentucky (26.74), Vermont (23.95), and Mississippi (20.83). Interestingly, Hawaii, Utah, and Massachusetts reported the lowest AAMRs—all under 10, at 6.69, 7.33, and 7.99, respectively (Supporting Table 5).

**FIGURE 6:**
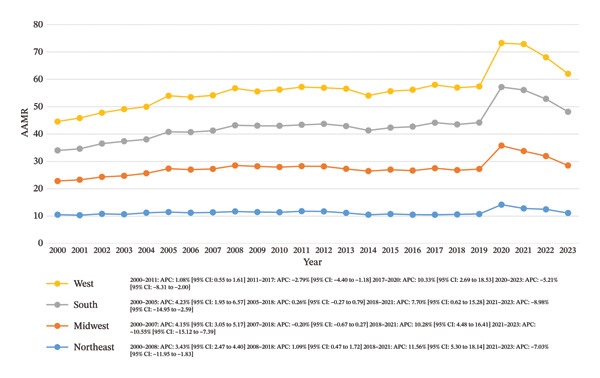
COPD‐related age‐adjusted mortality rates per 100,000 stratified by regions in adults (≥ 25 Years) with diabetes mellitus in the United States, 2000 to 2023. AAMR = age‐adjusted mortality rate; APC = annual percentage change.

Regionally, the Midwest had the highest AAMR at 16.30 per hundred thousand population, followed by South (15.51), West (13.22), and North East (11.28) (Supporting Table 7 and Figure 6).

## 4. Discussion

Diabetes is a complicated global condition that leads to a variety of chronic medical conditions such as cardiovascular diseases, kidney failure, and pulmonary complications. Recent medical literature has added significantly to the mortality data due to the coexistence of diabetes and COPD. The current study presents a comprehensive analysis of mortality trends from 2000 to 2023 among individuals with DM and coexisting COPD. It gave several important findings (Central illustration 1).

Over the past two decades, the overall mortality has gradually increased, with males having consistently higher mortality rates than females. Considering racial groups, NH American Indians were at the highest risk of death from these comorbidities, followed by NH African American and NH White adults. Also, from a geographical perspective, the Midwest region and rural areas recorded higher mortality rates than others.

The AAMR trends for COPD and DM showed a steady rise. Between 2000 and 2008, AAMR rapidly increased, followed by a plateau period from 2008 to 2018. However, a steeper increase in AAMR was observed during 2018–2021, after which mortality declined in 2022 and 2023. The identification of 2018 as a Joinpoint should be interpreted cautiously and does not necessarily indicate a single discrete change in that year. Rather, it may reflect the onset of a steeper upward trend against a prepandemic background of increasing chronic disease burden. National data have shown that COPD prevalence rose significantly through 2018, while diabetes prevalence among U.S. adults also increased over the same broad period, making it plausible that the observed inflection reflects accumulating multimorbidity in this high‐risk population before the additional impact of COVID‐19. The study of a cohort from the Taiwan Longitudinal Health Insurance Database between 2000 and 2013 also concluded that diabetic patients presenting with respiratory illnesses had worse mortality outcomes with reported hazard ratio (HR) 1.244 and HR 1.4–1.8 for patients with pre‐existing diabetes and incident (new onset) diabetes, respectively [14]. Similarly, a Swedish register–based cohort study of 5,624,306 individuals also reported a 272% higher risk of death from both COPD and type 2 diabetes, which is way greater than the risk of mortality from individual risks (COPD or diabetes alone) [15]. The sharp rise in AAMR observed between 2018 and 2021 was most plausibly driven by the COVID‐19 pandemic, given the well‐documented vulnerability of patients with chronic cardiopulmonary and metabolic disease during this period [16, 17]. However, other factors may also have contributed to the observed increase, including healthcare disruption, delayed presentations, reduced outpatient follow‐up, and interruptions in chronic disease management. The decline after 2021 most likely reflects a reduction in the acute impact of the COVID‐19 pandemic, along with the restoration of routine care for chronic conditions such as COPD and diabetes. Nonetheless, improved disease management and changes in the composition of the at‐risk population may also have contributed.

Emerging evidence and latest research strongly suggest a pathophysiological link between COPD and DM. In patients with T1DM, lung function abnormalities such as reduced total lung capacity (TLC), decreased diffusing capacity of the lung for carbon monoxide (DLCO), impaired elastic recoil with lesser end‐expiratory volume were observed [18]. A comparative study also proved how diabetic patients, even those not predisposed to any pulmonary problems, had stiff lungs with inefficient gaseous transport across alveoli, suggesting that lungs may be a target organ affected by diabetes [19, 20]. Diabetic and nondiabetic cohorts were compared on lung health and function upon exposure to hypoxia, hypercapnia, and exercise, which concluded that approximately 25% of people with diabetes exhibit a diminished ventilatory response to low oxygen or elevated carbon dioxide level [21].

Moreover, with mismanagement and worsening of blood glucose and severity of diabetes, vital lung functions like FVC and FEV1 were compromised [22–24]. This shows how early intervention in T2DM, through lifestyle changes and glucose‐lowering therapies, could reduce COPD risk.

Furthermore, both COPD and diabetes are deteriorating chronic inflammatory conditions where diabetes amplifies the inflammatory load in a lung injury. This is mainly due to elevated markers like C‐reactive protein (CRP), interleukin‐6 (IL‐6), and TNF‐alpha. High CRP levels, particularly, are linked to lower baseline FEV1 and FVC, highlighting their role in COPD and diabetes progression [25, 26]. Also, when blood glucose levels stay high for a long time in diabetes, it causes the body to produce harmful molecules like reactive oxygen species (ROS) and advanced glycation end products (AGEs). These AGEs bind to specific receptors called RAGE on the cells of lungs, which sets off stress and inflammation [27]. Over time, this damages important structural proteins like collagen and elastin, which help the lungs stay flexible and work properly [28].

Lastly, DM has specifically shown a decline in alveolar microvasculature reserves leading to impairments in lung size, blood perfusion, and capillary function [29].

This analysis identified that the AAMR associated with diabetes and COPD was significantly higher in males than in females. This finding is consistent with most of the previous literature, although recent trends have shown women to be at a greater risk of COPD as they are more susceptible to the harmful effects of smoking, tobacco, and air pollution on lung tissue [30]. Women generally have longer life expectancy, which may partly influence observed sex‐specific mortality patterns. Men overall have had higher COPD‐related mortality, which may partly reflect higher rates of tobacco [31] and alcohol use, both of which are major risk factors for COPD [32]. Also, men are often less proactive than women in seeking medical help due to a variety of factors, including societal expectations, and many men who smoke do not want to admit it to a doctor, which leads to improper diagnosis and poor health outcomes [33]. Moreover, estrogen in women may offer some protective effects against inflammation and oxidative stress, both of which are central to the pathophysiology of COPD and diabetes [34]. Again, men have more mortality due to higher DM prevalence [35] due to insulin resistance, and lifestyle factors like higher visceral fat and, as mentioned before, attitude toward health [36]. Through these findings, we can develop tailored prevention strategies that help to manage the elevated risk of COPD and diabetes in men.

Racial disparities were among one of the most striking outcomes of this study. Non‐Hispanic American Indian or Alaska Native populations consistently exhibited the highest AAMRs throughout the 23‐year observation period. The increased AAMRs among American Indian or Alaska Native populations likely reflect not only multimorbidity and barriers to pulmonary care but also broader conditions that influence prevention, diagnosis, and long‐term disease management [37]. Contributing factors may include socioeconomic disadvantage, geographic isolation, under‐resourced healthcare services, and the enduring effects of historical marginalization [38]. These disparities should therefore be interpreted in the context of unequal access to health‐promoting resources rather than individual behaviors alone. After NH American Indians, the consistently high mortality rates observed in the NH Black population may similarly reflect a greater burden of cardiometabolic risk factors, including obesity and diabetes, together with persistent barriers to preventive care, inequities in chronic disease management, and the adverse effects of systemic racism within healthcare systems [39]. NH White adults also showed comparably elevated mortality, although slightly lower than that observed in NH Black adults. This could be attributed to this group having a larger proportion of older adults compared to other racial groups [40]. Our study reveals a link between rising DM rates and increased COPD mortality, highlighting the need to identify and address the underlying causes and disparities in DM among older adults of distinct ethnicities.

From a geographic perspective, the Midwestern and Southern United States consistently bore the highest AAMRs across the study period. The marked state‐level variation likely reflects a clustering of differences in smoking burden, obesity and diabetes prevalence, rural residence, and access to coordinated chronic disease care, rather than a single explanatory factor, which may help explain why high‐burden states such as Oklahoma experience substantially greater mortality than lower‐burden states such as Hawaii [41]. Additionally, rural areas showed significantly higher death rates than urban areas. This has been proven previously when COPD and DM were individually studied in urban–rural settings [42, 43]. The rural–urban gap likely reflects differences in healthcare access, continuity of chronic disease management, delayed access to care, and underlying socioeconomic disadvantage. Limited availability of specialist services, greater distance from healthcare facilities, and reliance on telemedicine in geographically underserved settings may further affect the timeliness and quality of care, particularly for patients requiring regular follow‐up for both COPD and diabetes [44–47]. The COVID‐19 pandemic made these gaps even worse, with rural areas seeing their death rates rise at twice the rate of those in cities.

Individuals aged 65 and older faced the highest mortality rates during the study, which highlights how susceptible the older population is to COPD and DM. The reasons can be multiple and vary from patient to patient; these include respiratory complications (like exacerbations, pneumonia, and lung cancer), cardiovascular issues, and increased susceptibility to infections in the later stages of COPD. Diabetes further worsens the conditions as hyperglycemia impairs immune function, damages blood vessels, and causes mucus overproduction in the airways [48]. Both COPD and diabetes can present with cardiovascular complications [49] like coronary artery diseases [50], heart failure, arrhythmias, and pulmonary hypertension due to common risk factors like hypertension, high cholesterol, and severe COPD, which can cause Cor pulmonale. Lastly, age‐related impairment in physical function and frailty can exacerbate the impact of these chronic conditions and make it harder to manage.

These findings highlight the substantial mortality burden associated with coexisting DM and COPD and the need for more targeted, evidence‐based interventions. DM has exacerbated COPD‐related deaths, although mortality trends due to COPD alone were decreasing in the United States. This underscores the urgent need for coordinated evidence‐based strategies across clinical, public health, and policy sectors. With disparities in mind, males, NH American Indians, Southern district, and rural area population must have focused, equity‐driven interventions. Clinically, integrated COPD‐diabetes care models may offer the most directly relevant strategy for reducing mortality in this comorbid population. Such models are likely to be more effective than generic disease‐specific approaches because they address exacerbation prevention, glycemic control, medication coordination, and continuity of follow‐up simultaneously [51]. Although smoking cessation support, optimized diabetes monitoring, evidence‐based treatment, and improved access to care remain important, these measures are likely to have the greatest impact when embedded within integrated COPD–diabetes care models and access‐focused strategies for rural and underserved populations [52–55]. In the context of the disparities observed here, priority should be given to integrated care delivery, rural access interventions, and equity‐focused outreach in high‐burden populations.

Policy measures that strengthen telehealth access, improve chronic disease follow‐up, and expand healthcare availability in underserved settings may further help reduce excess mortality. These strategies are likely to be most effective when aligned with integrated COPD–diabetes care and targeted outreach in high‐burden populations.

### 4.1. Limitations

The present study should be viewed considering certain limitations. Although CDC WONDER database provided comprehensive access to U.S. mortality records, it lacks data of nonresidents and deaths that take place outside the United States. Secondly, because this analysis relied on CDC WONDER mortality records and ICD‐10 coding, it lacked patient‐level clinical information such as COPD severity, spirometric parameters, exacerbation burden, glycemic control, diabetes duration, medication use, treatment adherence, and cause‐specific care patterns. As a result, we were unable to determine whether the observed mortality patterns were driven by more advanced pulmonary disease, poorer metabolic control, treatment differences, or variation in access to guideline‐concordant care. Thirdly, we could not assess socioeconomic status or other contextual determinants because these variables are not available in the dataset. Future studies linking mortality records with clinical registries, electronic health records, or claims‐based cohorts are needed to clarify the clinical and healthcare‐system factors driving these mortality patterns, including differences according to GOLD stage, HbA1c, comorbidity burden, and treatment intensity.

## 5. Conclusion

Our study demonstrates a substantial increase in COPD‐ and DM‐related mortality among adults in the United States between 2000 and 2023. The burden was greatest among men, Non‐Hispanic American Indian populations, and rural communities. Demographic and regional variations demonstrated higher age‐adjusted mortality in rural settings than in urban areas. All this highlights the need to prioritize integrated COPD–diabetes care and equity‐focused public health strategies to reduce mortality in high‐burden populations.

**Nomenclature**
COPD: Chronic Obstructive Pulmonary Disease
DM: Diabetes Mellitus
AAMR: Age‐Adjusted Mortality Rates
AAPC: Average Annual Percentage Changes
APC: Annual Percent Change
NH: Non‐Hispanic
CDC‐WONDER: Centers for Disease Control and Prevention’s Wide‐ranging Online Data for Epidemiologic Research

## Funding

No funding was received for this manuscript.

## Ethics Statement

The authors have nothing to report.

## Consent

The authors have nothing to report.

## Conflicts of Interest

The authors have nothing to report.

## Supporting Information

Additional supporting information can be found online in the Supporting Information section.

## Supporting information


**Supporting Information** Supporting Table 1. Overall and sex‐stratified COPD‐related mortality among U.S. adults with DM from 2000 to 2023. Supporting Table 2: Annual percent change (APC) of COPD‐related age‐adjusted mortality rates per 100,000 in adults with diabetes mellitus in the United States, 2000 to 2023. Supporting Table 3. Overall and sex‐stratified COPD‐related AAMR in patients with DM per 100,000 in the United States from 2000 to 2023. Supporting Table 4. COPD‐ and DM‐related AAMR per 100,000 stratified by race in the United States from 2000 to 2023. Supporting Table 5. COPD‐ and DM‐related AAMR per 100,000 stratified by urbanization in the United States from 2000 to 2023. Supporting Table 6. COPD‐ and DM‐related AAMR per 100,000 stratified by states in the United States from 2000 to 2023. Supporting Table 7. COPD‐ and DM‐related AAMR per 100,000 stratified by census region in the United States from 2000 to 2023. Supporting Table 8. COPD‐ and DM‐related AAMR per 100,000 stratified by age group in the United States from 2000 to 2023.

## Data Availability

The data that support the findings of this study are available in the supporting information of this article.
