# Supplementary material for: Mortality Trends in Patients Aged 25 and Older With Comorbid COPD and Diabetes Mellitus in the United States: A CDC WONDER Database Study From 2000 to 2023
Source: Can Respir J. 2026 Jul 27;2026:3655829. doi: 10.1155/carj/3655829 (PMC13403471; doi:10.1155/carj/3655829)
Supplement: Supplementary file 1 — Supporting Information Supporting Table 1. Overall and sex‐stratified COPD‐related mortality among U.S. adults with DM from 2000 to 2023. Supporting Table 2: Annual percent change (APC) of COPD‐related age‐adjusted mortality rates per 100,000 in adults with diabetes mellitus in the United States, 2000 to 2023. Supporting Table 3. Overall and sex‐stratified COPD‐related AAMR in patients with DM per 100,000 in the United States from 2000 to 2023. Supporting Table 4. COPD‐ and DM‐related AAMR per 100,000 stratified by race in the United States from 2000 to 2023. Supporting Table 5. COPD‐ and DM‐related AAMR per 100,000 stratified by urbanization in the United States from 2000 to 2023. Supporting Table 6. COPD‐ and DM‐related AAMR per 100,000 stratified by states in the United States from 2000 to 2023. Supporting Table 7. COPD‐ and DM‐related AAMR per 100,000 stratified by census region in the United States from 2000 to 2023. Supporting Table 8. COPD‐ and DM‐related AAMR per 100,000 stratified by age group in the United States from 2000 to 2023. [file CARJ-2026-3655829-s001.docx]

**Supplementary Table 1. Overall and sex stratified COPD-related mortality among United States adults with DM from 2000 to 2023.**

| Year | Overall | Female | Male | Hispanic or Latino | NH American Indian | NH Black | NH White | NH Asian | Population |
| --- | --- | --- | --- | --- | --- | --- | --- | --- | --- |
| 2000 | 20010 | 9299 | 10711 | 723 | 94 | 1765 | 17174 | 200 | 181984640 |
| 2001 | 20869 | 9671 | 11198 | 855 | 119 | 1761 | 17853 | 233 | 184305128 |
| 2002 | 22197 | 10243 | 11954 | 918 | 112 | 1982 | 18870 | 257 | 186208028 |
| 2003 | 23199 | 10717 | 12482 | 940 | 133 | 2063 | 19736 | 266 | 188090429 |
| 2004 | 23842 | 11052 | 12790 | 992 | 152 | 2031 | 20370 | 249 | 190205384 |
| 2005 | 26260 | 12209 | 14051 | 1146 | 144 | 2362 | 22252 | 309 | 192551384 |
| 2006 | 26608 | 12438 | 14170 | 1157 | 162 | 2344 | 22506 | 374 | 195019359 |
| 2007 | 27477 | 12804 | 14673 | 1191 | 175 | 2469 | 23262 | 334 | 197403777 |
| 2008 | 29404 | 13594 | 15810 | 1266 | 195 | 2718 | 24795 | 374 | 199795090 |
| 2009 | 29542 | 13564 | 15978 | 1296 | 211 | 2830 | 24758 | 400 | 202107016 |
| 2010 | 30406 | 13962 | 16444 | 1383 | 211 | 2883 | 25437 | 427 | 203891983 |
| 2011 | 31713 | 14761 | 16952 | 1471 | 246 | 2978 | 26503 | 450 | 206592936 |
| 2012 | 32535 | 14645 | 17890 | 1682 | 270 | 3155 | 26923 | 438 | 208826037 |
| 2013 | 33375 | 15185 | 18190 | 1735 | 245 | 3228 | 27612 | 481 | 211085314 |
| 2014 | 32739 | 14712 | 18027 | 1668 | 301 | 3212 | 26975 | 478 | 213809280 |
| 2015 | 34632 | 15658 | 18974 | 1773 | 312 | 3407 | 28442 | 546 | 216553817 |
| 2016 | 35918 | 16108 | 19810 | 2020 | 330 | 3844 | 29078 | 526 | 218641417 |
| 2017 | 38110 | 17140 | 20970 | 2100 | 388 | 3973 | 30915 | 591 | 221447331 |
| 2018 | 38589 | 17212 | 21377 | 2133 | 377 | 4177 | 31127 | 646 | 223311190 |
| 2019 | 39878 | 17519 | 22359 | 2227 | 408 | 4279 | 32254 | 597 | 224981167 |
| 2020 | 51795 | 22934 | 28861 | 3475 | 526 | 6413 | 40338 | 919 | 226635013 |
| 2021 | 51411 | 22717 | 28694 | 2968 | 494 | 5896 | 40869 | 727 | 228238412 |
| 2022 | 49896 | 22260 | 27636 | 2833 | 447 | 5591 | 39820 | 730 | 229508599 |
| 2023 | 46352 | 20453 | 25899 | 2507 | 428 | 5211 | 36993 | 689 | 231529762 |
| Total | **796757** | **360857** | **435900** | **40459** | **6480** | **80572** | **654862** | **11241** | **4982722493** |

**Supplemental Table 2:** **Annual percent change (APC) of COPD-related Age-Adjusted Mortality Rates per 100,000 in Adults with Diabetes Mellitus in the United States, 2000 to 2023.**

| **Year Interval** | **APC (95% CI)** | **P-value** |
| --- | --- | --- |
| **Overall** | | |
| 2000-2008 | 2.9581(1.9204-5.8027) | < 0.000001 |
| 2008–2018 | 0.1843 (-2.2951 to 0.7667) | 0.791442 |
| 2018–2021 | 10.5675* (7.1149 to 13.1355) | < 0.000001 |
| 2021–2023 | -9.2077* (-12.7770 to -5.9006) | < 0.000001 |
| **Men** | | |
| 2000–2008 | 2.4196* (1.4039 to 6.1322) | 0.000400 |
| 2008–2018 | 2.4196* (1.4039 to 6.1322) | 0.000400 |
| 2018–2021 | 10.4104* (7.0943 to 12.8587) | < 0.000001 |
| 2021–2023 | -9.9700* (-12.4593 to -5.4781) | < 0.000001 |
| **Women** | | |
| 2000–2007 | 3.7137* (2.2683 to 7.3496) | 0.000400 |
| 2007–2018 | 0.2163 (-2.2510 to 0.7894) | 0.722655 |
| 2018–2021 | 10.2023* (6.4070 to 12.7493) | 0.000400 |
| 2021–2023 | -8.3289* (-12.6325 to -3.8749) | 0.001600 |
| **NH White** | | |
| 2000–2008 | 3.2688* (2.4019 to 4.8992) | < 0.000001 |
| 2008–2018 | 0.3417 (-0.9452 to 0.8592) | 0.379524 |
| 2018–2021 | 10.3593* (7.4695 to 12.2536) | < 0.000001 |
| 2021–2023 | -7.4319* (-10.7046 to -4.4404) | < 0.000001 |
| **NH Black** | | |
| 2000–2018 | 1.5269* (0.5672 to 2.1822) | 0.013197 |
| 2018–2021 | 12.7092* (6.2896 to 16.8862) | 0.007998 |
| 2021–2023 | -11.2458* (-17.7642 to -2.9488) | 0.016797 |
| **NH American Indian** | | |
| 2000–2021 | 3.4511* (3.0047 to 5.9522) | 0.001200 |
| 2021–2023 | -7.6042 (-14.8887 to 2.3765) | 0.143171 |

NH Asian

| 2000–2023 | -0.2832 (-0.9625 to 0.5711) | 0.583083 |
| --- | --- | --- |
| **Hispanic** | | |
| 2000–2017 | -0.2557 (-4.2859 to 0.7367) | 0.508698 |
| 2017–2020 | 10.9449* (2.9138 to 15.4290) | 0.011598 |
| 2020–2023 | -9.5135* (-20.1384 to -4.1335) | 0.016397 |
| **Rural areas** | | |
| 2000–2007 | 4.1771* (2.9240 to 7.4539) | 0.000001 |
| 2007–2018 | 1.3726 (-0.7003 to 1.9067) | 0.087084 |
| 2018–2020 | 13.6740* (7.5550 to 17.0918) | < 0.000001 |
| **Urban areas** | | |
| 2000–2008 | 2.8096* (1.6266 to 6.2566) | 0.000400 |
| 2008–2018 | -1.1973 (-4.2696 to 0.5168) | 0.485903 |
| 2018–2020 | 12.3749* (4.6036 to 16.4536) | < 0.000001 |
| **Northeast region** | | |
| 2000–2011 | 1.0791* (0.5464 to 1.6147) | 0.000734 |
| 2011–2017 | -2.7912* (-4.3779 to -1.1763) | 0.026010 |
| 2017–2021 | 10.3305* (2.6940 to 18.5349) | 0.011035 |
| 2021–2023 | -5.2099* (-8.3066 to -2.0086) | 0.004066 |
| **South region** | | |
| 2000–2013 | 3.4325* (2.4753 to 4.3986) | 0.000003 |
| 2013–2018 | 1.0928* (0.4658 to 1.7236) | 0.0021038 |
| 2018–2021 | 11.5575* (5.3048 to 18.1814) | 0.001262 |
| 2021–2023 | -7.0275* (-11.9467 to -1.8335) | 0.012508 |
| **Midwest region** | | |
| 2000–2013 | 4.8152* (3.2051 to 5.1672) | < 0.000001 |
| 2013–2018 | -0.1972 (-0.6703 to 0.2781) | 0.385659 |
| 2018–2021 | 10.2844* (4.4827 to 16.4082) | 0.001781 |
| 2021–2023 | -11.5528* (-15.1207 to -5.7391) | 0.000550 |
| **West region** | | |
| 2000–2013 | 4.2338* (1.9484 to 6.5704) | 0.000140 |
| 2013–2018 | 0.2597 (-0.2221 to 0.7439) | 0.265668 |
| 2018–2021 | 7.6980* (0.6190 to 15.2750) | 0.034805 |
| 2021–2023 | -8.9817* (-14.9496 to -2.5949) | 0.0102080 |
| APC = annual percent change; NH = non-Hispanic; * Indicates that the annual percentage change (APC) is significantly different from zero at α = 0.05. AAMR = age-adjusted mortality rate. The data for urbanization is only available till 2020 in the CDC Database. | | |

**Supplementary Table 3. Overall and sex stratified COPD-related AAMR in patients with DM per 100,000 in the United States from 2000-2023**

| Year | Overall | Female | Male |
| --- | --- | --- | --- |
| 2000 | 11.19 [11.03–11.34] | 8.84 [8.66–9.02] | 14.95 [14.66–15.24] |
| 2001 | 11.49 [11.33–11.64] | 9.06 [8.88–9.24] | 15.30 [15.01–15.59] |
| 2002 | 12.04 [11.88–12.19] | 9.49 [9.31–9.68] | 16.01 [15.72–16.30] |
| 2003 | 12.38 [12.22–12.54] | 9.79 [9.61–9.98] | 16.37 [16.08–16.67] |
| 2004 | 12.52 [12.36–12.68] | 9.99 [9.80–10.18] | 16.38 [16.09–16.67] |
| 2005 | 13.55 [13.38–13.71] | 10.87 [10.68–11.07] | 17.59 [17.30–17.89] |
| 2006 | 13.46 [13.30–13.62] | 10.93 [10.73–11.12] | 17.38 [17.09–17.68] |
| 2007 | 13.67 [13.50–13.83] | 11.06 [10.86–11.25] | 17.54 [17.25–17.83] |
| 2008 | 14.32 [14.15–14.48] | 11.53 [11.33–11.72] | 18.47 [18.18–18.76] |
| 2009 | 14.13 [13.97–14.29] | 11.31 [11.12–11.50] | 18.15 [17.87–18.44] |
| 2010 | 14.27 [14.11–14.43] | 11.46 [11.27–11.65] | 18.34 [18.05–18.62] |
| 2011 | 14.50 [14.34–14.66] | 11.83 [11.64–12.02] | 18.33 [18.05–18.61] |
| 2012 | 14.48 [14.32–14.64] | 11.50 [11.31–11.69] | 18.73 [18.45–19.01] |
| 2013 | 14.44 [14.28–14.60] | 11.66 [11.48–11.85] | 18.40 [18.13–18.68] |
| 2014 | 13.82 [13.67–13.97] | 11.00 [10.82–11.18] | 17.68 [17.41–17.94] |
| 2015 | 14.24 [14.09–14.39] | 11.46 [11.28–11.64] | 18.05 [17.79–18.31] |
| 2016 | 14.43 [14.28–14.58] | 11.55 [11.37–11.74] | 18.32 [18.06–18.58] |
| 2017 | 14.94 [14.79–15.09] | 12.02 [11.84–12.21] | 18.87 [18.61–19.13] |
| 2018 | 14.74 [14.59–14.89] | 11.82 [11.64–12.00] | 18.67 [18.41–18.92] |
| 2019 | 14.86 [14.71–15.01] | 11.71 [11.53–11.88] | 19.05 [18.80–19.30] |
| 2020 | 18.91 [18.74–19.07] | 15.06 [14.86–15.26] | 24.03 [23.74–24.31] |
| 2021 | 19.03 [18.86–19.20] | 15.24 [15.04–15.44] | 24.00 [23.71–24.28] |
| 2022 | 17.78 [17.62–17.93] | 14.32 [14.13–14.52] | 22.39 [22.12–22.66] |
| 2023 | 16.28 [16.13–16.43] | 13.06 [12.88–13.24] | 20.54 [20.29–20.80] |

**Supplementary Table 4. COPD and DM related AAMR per 100,000 stratified by Race in the United**

| Year | Hispanic | NH American Indian | NH Black | NH white | NH Asian |
| --- | --- | --- | --- | --- | --- |
| 2000 | 8.64 [7.99–9.29] | 12.46 [9.98–15.37] | 11.80 [11.24–12.36] | 11.46 [11.29–11.63] | 5.22 [4.47–5.97] |
| 2001 | 9.57 [8.91–10.23] | 14.79 [12.01–17.56] | 11.59 [11.04–12.14] | 11.80 [11.62–11.97] | 5.62 [4.88–6.36] |
| 2002 | 9.76 [9.11–10.41] | 14.54 [11.73–17.34] | 12.75 [12.18–13.31] | 12.33 [12.15–12.51] | 5.71 [4.99–6.43] |
| 2003 | 9.53 [8.90–10.16] | 15.46 [12.67–18.24] | 13.05 [12.48–13.62] | 12.76 [12.58–12.93] | 5.58 [4.89–6.27] |
| 2004 | 9.48 [8.87–10.09] | 16.94 [14.10–19.79] | 12.53 [11.98–13.09] | 13.05 [12.87–13.23] | 4.92 [4.30–5.55] |
| 2005 | 10.18 [9.57–10.79] | 15.41 [12.74–18.08] | 14.22 [13.64–14.81] | 14.05 [13.87–14.24] | 5.79 [5.13–6.45] |
| 2006 | 9.95 [9.36–10.54] | 16.86 [14.10–19.63] | 13.68 [13.11–14.24] | 14.05 [13.87–14.24] | 6.60 [5.91–7.28] |
| 2007 | 9.58 [9.02–10.14] | 18.41 [15.51–21.30] | 14.08 [13.51–14.65] | 14.29 [14.10–14.47] | 5.50 [4.90–6.10] |
| 2008 | 9.73 [9.18–10.28] | 18.42 [15.67–21.18] | 15.11 [14.52–15.69] | 15.02 [14.84–15.21] | 5.91 [5.30–6.52] |
| 2009 | 9.45 [8.92–9.99] | 19.99 [17.13–22.86] | 15.28 [14.70–15.86] | 14.77 [14.58–14.95] | 6.10 [5.50–6.71] |
| 2010 | 9.80 [9.27–10.33] | 19.48 [16.67–22.29] | 15.13 [14.57–15.70] | 14.97 [14.78–15.15] | 6.09 [5.50–6.68] |
| 2011 | 9.62 [9.12–10.13] | 21.55 [18.69–24.41] | 15.10 [14.55–15.66] | 15.30 [15.12–15.49] | 6.00 [5.44–6.57] |
| 2012 | 10.34 [9.83–10.85] | 22.75 [19.90–25.61] | 15.37 [14.82–15.92] | 15.20 [15.02–15.39] | 5.33 [4.82–5.84] |
| 2013 | 10.25 [9.76–10.74] | 18.92 [16.42–21.41] | 15.14 [14.60–15.68] | 15.31 [15.13–15.50] | 5.53 [5.03–6.03] |
| 2014 | 9.11 [8.66–9.56] | 21.97 [19.36–24.58] | 14.48 [13.96–15.00] | 14.70 [14.52–14.88] | 5.13 [4.66–5.59] |
| 2015 | 9.12 [8.68–9.55] | 21.61 [19.10–24.12] | 14.79 [14.28–15.30] | 15.17 [14.99–15.35] | 5.33 [4.88–5.79] |
| 2016 | 9.89 [9.45–10.33] | 21.61 [19.18–24.05] | 16.07 [15.55–16.60] | 15.34 [15.16–15.52] | 4.85 [4.43–5.27] |
| 2017 | 9.69 [9.26–10.12] | 25.30 [22.67–27.92] | 16.06 [15.55–16.58] | 15.92 [15.74–16.10] | 5.15 [4.73–5.57] |
| 2018 | 9.42 [9.01–9.83] | 23.36 [20.92–25.80] | 16.37 [15.86–16.88] | 15.69 [15.52–15.87] | 5.38 [4.96–5.80] |
| 2019 | 9.41 [9.01–9.81] | 23.92 [21.51–26.32] | 16.25 [15.75–16.75] | 15.97 [15.79–16.14] | 4.73 [4.34–5.11] |
| 2020 | 14.11 [13.63–14.59] | 29.52 [26.92–32.12] | 23.74 [23.14–24.34] | 19.68 [19.48–19.87] | 6.88 [6.43–7.33] |
| 2021 | 11.86 [11.42–12.30] | 30.48 [27.70–33.26] | 22.07 [21.49–22.66] | 20.54 [20.34–20.74] | 5.80 [5.37–6.23] |
| 2022 | 10.74 [10.33–11.15] | 25.77 [23.33–28.22] | 20.38 [19.83–20.93] | 19.26 [19.07–19.46] | 5.33 [4.94–5.72] |
| 2023 | 9.25 [8.87–9.62] | 24.15 [21.82–26.49] | 18.69 [18.17–19.22] | 17.79 [17.61–17.98] | 4.84 [4.47–5.20] |

**Supplementary Table 5. COPD and DM related AAMR per 100,000 stratified by Urbanization in the United States from 2000-2023.**

| Year | Urban areas | Rural areas |
| --- | --- | --- |
| 2000 | 10.67 [10.50–10.84] | 13.37 [12.98–13.76] |
| 2001 | 10.81 [10.64–10.97] | 14.47 [14.06–14.87] |
| 2002 | 11.31 [11.14–11.48] | 15.20 [14.79–15.61] |
| 2003 | 11.54 [11.37–11.71] | 16.05 [15.63–16.47] |
| 2004 | 11.78 [11.61–11.95] | 15.80 [15.39–16.22] |
| 2005 | 12.65 [12.47–12.83] | 17.57 [17.13–18.01] |
| 2006 | 12.65 [12.47–12.82] | 17.25 [16.82–17.68] |
| 2007 | 12.70 [12.53–12.87] | 18.10 [17.66–18.53] |
| 2008 | 13.38 [13.21–13.56] | 18.68 [18.24–19.12] |
| 2009 | 13.01 [12.84–13.19] | 19.13 [18.68–19.57] |
| 2010 | 13.26 [13.09–13.43] | 18.99 [18.55–19.43] |
| 2011 | 13.43 [13.26–13.60] | 19.54 [19.09–19.98] |
| 2012 | 13.29 [13.13–13.46] | 20.17 [19.72–20.62] |
| 2013 | 13.33 [13.17–13.50] | 19.85 [19.41–20.29] |
| 2014 | 12.66 [12.50–12.82] | 19.26 [18.83–19.70] |
| 2015 | 12.94 [12.78–13.10] | 20.62 [20.18–21.07] |
| 2016 | 13.21 [13.05–13.37] | 20.79 [20.35–21.23] |
| 2017 | 13.57 [13.41–13.72] | 21.71 [21.26–22.16] |
| 2018 | 13.36 [13.20–13.51] | 21.74 [21.29–22.18] |
| 2019 | 13.30 [13.14–13.45] | 22.92 [22.47–23.38] |
| 2020 | 17.09 [16.91–17.26] | 28.27 [27.76–28.77] |

**Supplementary Table 6. COPD and DM related AAMR per 100,000 stratified by States in the United States from 2000-2023.**

| State | Age Adjusted Rate |
| --- | --- |
| Alabama | 12.65 [12.39–12.91] |
| Alaska | 12.01 [11.11–12.91] |
| Arizona | 8.81 [8.62–8.99] |
| Arkansas | 15.04 [14.69–15.40] |
| California | 13.38 [13.27–13.48] |
| Colorado | 15.37 [15.06–15.68] |
| Connecticut | 9.48 [9.23–9.73] |
| Delaware | 13.70 [13.09–14.30] |
| District of Columbia | 10.16 [9.46–10.86] |
| Florida | 10.37 [10.26–10.47] |
| Georgia | 10.80 [10.61–10.99] |
| Hawaii | 6.69 [6.35–7.03] |
| Idaho | 14.04 [13.54–14.54] |
| Illinois | 11.35 [11.19–11.50] |
| Indiana | 19.04 [18.76–19.32] |
| Iowa | 17.08 [16.72–17.43] |
| Kansas | 12.58 [12.25–12.92] |
| Kentucky | 26.74 [26.34–27.14] |
| Louisiana | 13.06 [12.78–13.34] |
| Maine | 15.79 [15.29–16.30] |
| Maryland | 12.98 [12.73–13.23] |
| Massachusetts | 7.99 [7.82–8.16] |
| Michigan | 15.37 [15.17–15.56] |
| Minnesota | 15.50 [15.23–15.78] |
| Mississippi | 20.83 [20.39–21.26] |
| Missouri | 15.41 [15.16–15.66] |
| Montana | 15.62 [15.02–16.23] |
| Nebraska | 18.45 [17.95–18.96] |
| Nevada | 9.56 [9.23–9.88] |
| New Hampshire | 14.77 [14.24–15.30] |
| New Jersey | 9.86 [9.69–10.02] |
| New Mexico | 11.33 [10.95–11.72] |
| New York | 9.47 [9.36–9.58] |
| North Carolina | 17.04 [16.82–17.26] |
| North Dakota | 16.60 [15.84–17.36] |
| Ohio | 21.13 [20.92–21.34] |
| Oklahoma | 28.79 [28.35–29.23] |
| Oregon | 17.29 [16.96–17.62] |
| Pennsylvania | 14.33 [14.17–14.49] |
| Rhode Island | 17.12 [16.51–17.73] |
| South Carolina | 15.96 [15.66–16.26] |
| South Dakota | 17.70 [16.98–18.41] |
| Tennessee | 19.70 [19.42–19.98] |
| Texas | 15.70 [15.56–15.85] |
| Utah | 7.33 [7.02–7.65] |
| Vermont | 23.95 [23.00–24.89] |
| Virginia | 11.11 [10.92–11.31] |
| Washington | 15.65 [15.39–15.90] |
| West Virginia | 28.53 [27.95–29.11] |
| Wisconsin | 13.72 [13.48–13.97] |
| Wyoming | 18.77 [17.80–19.73] |

**Supplementary Table 7. COPD and DM related AAMR per 100,000 stratified by Census Region in the United States from 2000-2023.**

| Year | Northeast | Midwest | South | West |
| --- | --- | --- | --- | --- |
| 2000 | 10.52 [10.19–10.85] | 12.30 [11.97–12.64] | 11.15 [10.89–11.41] | 10.59 [10.25–10.93] |
| 2001 | 10.33 [10.01–10.65] | 12.95 [12.61–13.29] | 11.31 [11.05–11.57] | 11.25 [10.91–11.60] |
| 2002 | 10.80 [10.48–11.13] | 13.51 [13.16–13.85] | 12.18 [11.91–12.45] | 11.29 [10.95–11.63] |
| 2003 | 10.63 [10.31–10.96] | 14.10 [13.75–14.45] | 12.61 [12.34–12.88] | 11.75 [11.40–12.09] |
| 2004 | 11.21 [10.87–11.54] | 14.44 [14.09–14.80] | 12.38 [12.12–12.65] | 11.93 [11.59–12.27] |
| 2005 | 11.46 [11.13–11.80] | 15.89 [15.52–16.26] | 13.42 [13.14–13.69] | 13.22 [12.86–13.57] |
| 2006 | 11.21 [10.88–11.54] | 15.78 [15.41–16.15] | 13.68 [13.41–13.95] | 12.78 [12.44–13.13] |
| 2007 | 11.34 [11.01–11.67] | 15.88 [15.52–16.25] | 14.00 [13.72–14.27] | 12.92 [12.57–13.27] |
| 2008 | 11.68 [11.34–12.01] | 16.84 [16.47–17.21] | 14.67 [14.40–14.95] | 13.50 [13.15–13.85] |
| 2009 | 11.47 [11.14–11.79] | 16.72 [16.35–17.09] | 14.83 [14.55–15.10] | 12.53 [12.20–12.86] |
| 2010 | 11.40 [11.07–11.73] | 16.52 [16.15–16.89] | 15.05 [14.78–15.33] | 13.21 [12.87–13.54] |
| 2011 | 11.77 [11.44–12.10] | 16.47 [16.11–16.83] | 15.12 [14.85–15.40] | 13.81 [13.47–14.15] |
| 2012 | 11.71 [11.39–12.04] | 16.46 [16.11–16.82] | 15.51 [15.24–15.78] | 13.19 [12.87–13.52] |
| 2013 | 11.16 [10.84–11.47] | 16.10 [15.75–16.45] | 15.63 [15.36–15.90] | 13.63 [13.31–13.96] |
| 2014 | 10.49 [10.18–10.79] | 15.97 [15.62–16.32] | 14.84 [14.58–15.10] | 12.72 [12.40–13.03] |
| 2015 | 10.76 [10.46–11.07] | 16.22 [15.87–16.57] | 15.32 [15.07–15.58] | 13.34 [13.02–13.65] |
| 2016 | 10.53 [10.23–10.83] | 16.12 [15.78–16.46] | 16.04 [15.78–16.30] | 13.44 [13.13–13.75] |
| 2017 | 10.50 [10.21–10.80] | 17.02 [16.67–17.37] | 16.61 [16.35–16.87] | 13.79 [13.48–14.10] |
| 2018 | 10.62 [10.33–10.91] | 16.17 [15.83–16.51] | 16.70 [16.44–16.95] | 13.45 [13.15–13.75] |
| 2019 | 10.75 [10.46–11.04] | 16.46 [16.12–16.79] | 16.96 [16.70–17.21] | 13.19 [12.90–13.48] |
| 2020 | 14.17 [13.83–14.50] | 21.56 [21.18–21.95] | 21.43 [21.14–21.71] | 16.07 [15.75–16.40] |
| 2021 | 12.79 [12.47–13.10] | 20.95 [20.56–21.33] | 22.33 [22.04–22.63] | 16.77 [16.44–17.10] |
| 2022 | 12.46 [12.15–12.77] | 19.45 [19.09–19.81] | 20.92 [20.64–21.19] | 15.22 [14.92–15.53] |
| 2023 | 11.11 [10.82–11.40] | 17.38 [17.04–17.72] | 19.62 [19.35–19.89] | 13.88 [13.59–14.18] |

**Supplementary Table 8. COPD and DM related AAMR per 100,000 stratified by Age Grp in the United States from 2000-2023.**

| Year | Young Adults  (25-44) | Middle-Aged Adults  (45-64) | Old Adults  (65-85+) |
| --- | --- | --- | --- |
| 2000 | 0.11 [0.09–0.13] | 5.06 [4.88–5.24] | 48.09 [47.36–48.81] |
| 2001 | 0.16 [0.14–0.19] | 5.06 [4.89–5.24] | 49.49 [48.75–50.22] |
| 2002 | 0.16 [0.14–0.19] | 5.20 [5.03–5.37] | 52.06 [51.31–52.80] |
| 2003 | 0.22 [0.18–0.25] | 5.50 [5.32–5.67] | 53.19 [52.43–53.94] |
| 2004 | 0.16 [0.14–0.19] | 5.68 [5.51–5.85] | 53.71 [52.96–54.47] |
| 2005 | 0.22 [0.19–0.25] | 6.15 [5.98–6.33] | 57.99 [57.21–58.76] |
| 2006 | 0.16 [0.14–0.19] | 6.11 [5.94–6.29] | 57.73 [56.96–58.51] |
| 2007 | 0.22 [0.19–0.25] | 6.30 [6.12–6.47] | 58.35 [57.58–59.12] |
| 2008 | 0.22 [0.19–0.25] | 6.51 [6.34–6.69] | 61.29 [60.51–62.07] |
| 2009 | 0.26 [0.23–0.30] | 6.64 [6.46–6.81] | 60.03 [59.26–60.79] |
| 2010 | 0.22 [0.19–0.25] | 6.78 [6.60–6.95] | 60.58 [59.81–61.34] |
| 2011 | 0.22 [0.19–0.25] | 6.78 [6.60–6.95] | 61.76 [60.99–62.52] |
| 2012 | 0.27 [0.24–0.31] | 6.84 [6.66–7.01] | 61.44 [60.69–62.19] |
| 2013 | 0.27 [0.24–0.31] | 7.13 [6.96–7.31] | 60.71 [59.97–61.45] |
| 2014 | 0.32 [0.28–0.36] | 6.93 [6.76–7.10] | 57.77 [57.06–58.47] |
| 2015 | 0.27 [0.23–0.31] | 7.19 [7.02–7.36] | 59.59 [58.88–60.30] |
| 2016 | 0.22 [0.19–0.25] | 7.73 [7.55–7.91] | 59.75 [59.04–60.45] |
| 2017 | 0.27 [0.23–0.31] | 7.84 [7.66–8.02] | 62.01 [61.31–62.72] |
| 2018 | 0.27 [0.24–0.31] | 7.84 [7.66–8.02] | 60.99 [60.30–61.68] |
| 2019 | 0.22 [0.19–0.25] | 7.78 [7.60–7.96] | 61.84 [61.16–62.53] |
| 2020 | 0.33 [0.29–0.37] | 9.68 [9.48–9.88] | 78.96 [78.20–79.73] |
| 2021 | 0.33 [0.29–0.37] | 10.05 [9.85–10.26] | 78.92 [78.15–79.69] |
| 2022 | 0.37 [0.33–0.42] | 9.40 [9.20–9.59] | 73.56 [72.84–74.29] |
| 2023 | 0.27 [0.23–0.31] | 8.31 [8.13–8.50] | 68.04 [67.35–68.73] |
